# *Xanthomonas* spp.: Devastating Plant Pathogens and Sustainable Management Strategies

**DOI:** 10.3390/pathogens15020175

**Published:** 2026-02-05

**Authors:** Kamran Shah, Yanbing Guo, Muhammad Adnan, Hongzhi Wu

**Affiliations:** 1 College of Landscape and Horticulture, Yunnan Agricultural University, Kunming 650201, China; yanbingguo@139.com (Y.G.); madnan@outlook.com (M.A.); 2 State Key Laboratory for Development and Utilization of Forest Food Resources, Zhejiang A&F University, Hangzhou 311300, China; 3 Zhejiang Key Laboratory of Non-wood Forest Products Quality Regulation and Processing Utilization, Zhejiang A&F University, Hangzhou 311300, China; 4 College of Horticulture, South China Agricultural University, Guangzhou 510642, China; 5 College of Horticulture, Northwest A&F University, Yangling 712100, China

**Keywords:** *Xanthomonas*, plant pathogens, disease management, genomic plasticity, sustainable agriculture

## Abstract

The genus *Xanthomonas* comprises devastating plant pathogens responsible for significant yield losses in globally critical crops such as rice (*Oryza sativa* L.), citrus (*Citrus* L. spp.), cassava (*Manihot esculenta* Crantz), and tomato (*Solanum lycopersicum* L.). This review synthesizes current knowledge on the molecular mechanisms driving *Xanthomonas* pathogenicity, including the type III secretion system (T3SS) that translocates effector proteins, transcription activator-like effectors (TALEs) that reprogram host transcription, and extracellular polysaccharides (EPS) that promote biofilm formation and immune evasion, which collectively enable host colonization, immune suppression, and disease progression. Rapid adaptation through genomic plasticity and horizontal gene transfer (HGT) exacerbates challenges in disease management by facilitating evasion of host defenses and environmental stressors. Economically, *Xanthomonas* spp. inflict billions in annual losses through crop damage, trade restrictions, and eradication efforts, disproportionately affecting resource-limited regions. Emerging antibiotic resistance and climate-driven shifts in pathogen distribution further threaten food security. Sustainable strategies, such as CRISPR-based genome editing to disrupt susceptibility genes, biocontrol agents (e.g., *Bacillus* and *Pseudomonas* spp.), and nanotechnology-driven antimicrobials offer promising alternatives to conventional copper-based and chemical controls. This review underscores the urgent need for integrated, climate-resilient management approaches to mitigate the ecological and socioeconomic impacts of *Xanthomonas* diseases, bridging genomic insights with innovative control measures, to address escalating threats posed by these pathogens in a changing global climate.

## 1. Introduction

The genus *Xanthomonas* comprises a phylogenetically diverse group of Gram-negative bacterial pathogens that collectively infect over 400 plant species, including globally critical crops such as rice (*Oryza sativa* L.), citrus (*Citrus* L. spp.), cassava (*Manihot esculenta* Crantz), and tomato (*Solanum lycopersicum* L.) [1]. These pathogens rely on multiple coordinated virulence mechanisms, including delivery of effector proteins through the T3SS, the action of secreted TALEs and other type III effectors, production of EPS and cell wall–degrading enzymes, and quorum sensing (QS) regulated biofilm formation, to colonize host tissues, suppress plant immunity, and induce disease symptoms such as leaf blight, cankers, and wilts [2,3]. Recent studies highlight the role of host–pathogen co-evolution in shaping *Xanthomonas* pathogenicity, with effectors such as AvrBsT and TALEs directly manipulating plant transcriptional machinery to promote bacterial survival [4,5]. Genomic plasticity, driven by HGT and recombination (including plasmids, integrative/conjugative elements, transposons, and genomic islands affecting effector repertoires, LPS loci, and metal-resistance determinants), enables rapid adaptation to host defenses and environmental stressors, complicating disease management [2,6]. Importantly, while the genus *Xanthomonas* is broad in host association when viewed in aggregate, most major diseases are caused by host-adapted species/pathovars with comparatively restricted host ranges; in practice, host specificity is frequently determined by effector complements (including TALEs) and other accessory loci that can vary among lineages due to recombination and HGT. Accordingly, throughout this review we refer to genome-resolved species names and pathovar/lineage designations when discussing host range, race structure, and management implications.

The economic burden of *Xanthomonas*-associated diseases is profound, with annual global losses estimated in the billions of dollars due to reduced crop yields, trade restrictions, and management costs [3]. For example, *Xanthomonas oryzae* pv. *oryzae*, the causal agent of bacterial blight in rice, destroys up to 75% of harvests in endemic regions, threatening food security for millions across Asia [3]. Similarly, citrus canker caused by *Xanthomonas citri* subsp. *citri* has triggered extensive orchard destruction and costly quarantine programs in the Americas [7]. Smallholder farmers in developing nations are disproportionately affected, as recurring outbreaks destabilize livelihoods and amplify poverty cycles [8,9]. Compounding these challenges, prolonged use of copper-based bactericides and antibiotics has driven the emergence of resistant strains, diminishing the efficacy of conventional controls [10].

*Xanthomonas* spp. pose systemic threats to agricultural sustainability through their capacity to infect staple crops and adapt to climate variability [6,11]. Field and microcosm studies further show that these bacteria can survive only briefly in uninfested native soils, but can persist for much longer periods when protected in infected crop debris and seeds remaining in the field between seasons [12,13]. Cassava bacterial blight (*Xanthomonas phaseoli* pv. *manihotis*) and banana *Xanthomonas* wilt (*Xanthomonas vasicola* pv. *musacearum*) exemplify diseases that disrupt regional food systems and economies, particularly in sub-Saharan Africa [8,9]. Environmental factors such as high humidity and temperature fluctuations further accelerate pathogen spread, complicating containment efforts [14]. The lack of durable resistance in commercial crop varieties, coupled with limited surveillance infrastructure in resource-poor regions, heightens vulnerability to epidemics [15,16]. Addressing these threats requires integrated strategies that combine host resistance, precision diagnostics, and eco-friendly antimicrobial agents to mitigate the ecological and socioeconomic impacts of *Xanthomonas* diseases [17,18]. This review synthesizes recent advances in understanding *Xanthomonas* biology, explores emerging sustainable management approaches, and outlines priorities for climate-resilient disease control.

Foundational work before the genomics era established the practical importance of seed/propagule health, sanitation, and early race concepts for managing bacterial diseases, while later molecular genetics and population genomics clarified how conserved virulence modules (e.g., T3SS, DSF signaling, EPS/LPS) and rapidly evolving effector repertoires jointly shape host adaptation and epidemic turnover. This historical progression helps explain why modern management increasingly combines classical phytosanitation with effector-informed resistance strategies and surveillance.

Given the breadth of the *Xanthomonas* genus, this review focuses on four pathosystems for which race structure, effector biology, and management strategies are comparatively well resolved: the two major foliar diseases of rice bacterial leaf blight (*X. oryzae* pv. *oryzae*) and bacterial leaf streak (*X. oryzae* pv. *oryzicola*), citrus canker (*X. citri* subsp. *citri*), black rot of brassicas (*X. campestris* pv. *campestris*), and bacterial spot of tomato and pepper (*X. euvesicatoria*). These diseases were selected because they (i) account for disproportionate yield losses in major staple or high-value crops, (ii) have served as model systems for dissecting gene-for-gene interactions and effector-triggered susceptibility (ETS) and immunity, and (iii) exemplify contrasting monocot and dicot pathosystems in both temperate and tropical production regions [2,3,15,19,20]. Other *Xanthomonas* diseases (e.g., on cassava, banana, walnut, and cereals) are discussed comparatively where they provide additional insight into population biology or management, but the primary emphasis throughout is on translating mechanistic knowledge from these four species into sustainable control strategies.

Available evidence indicates that cross-pathogenicity across the focal crop pathosystems is generally limited at the species/pathovar level (reflecting host-adapted effector repertoires and accessory genomes), whereas substantial within-pathosystem diversity (races/pathotypes and rapidly shifting effector profiles) is common and can erode single-gene resistance. Consequently, durable resistance typically benefits from stacking complementary mechanisms (e.g., NLR/executor genes plus recessive or edited susceptibility targets) and from monitoring local population structure. Similarly, biocontrol efficacy may vary over time and space; using multi-strain/consortium approaches and integrating biocontrol with host resistance and anti-virulence tactics can help reduce selection for escape variants. CRISPR-based promoter editing of susceptibility genes is a particularly promising route to durability when it targets multiple effector-binding sites and is deployed alongside surveillance to detect shifts in TALE repertoires.

To our knowledge, this is the first review to (i) treat bacterial leaf blight and bacterial leaf streak (BLS) of rice together as a twin *X. oryzae* leaf disease complex, (ii) systematically link race structure and effector repertoires to both classical and CRISPR-enabled resistance strategies across four model pathosystems, and (iii) embed these insights within a climate-change and antimicrobial-resistance framework. By integrating population genomics, effector biology, host resistance mechanisms, and sustainable management tools (biocontrol, nanoparticles, effector-targeted chemistries), we aim to provide a conceptual roadmap for designing climate-resilient, effector-informed control strategies against *Xanthomonas* diseases.

### 1.1. Origin, Discovery, and Historical Context

The genus *Xanthomonas* is hypothesized to have originated from ancestral environmental, primarily water-associated *Gammaproteobacteria*, evolving through HGT, recombination, and host specialization to become a globally significant group of plant pathogens [21,22,23]. Phylogenomic studies suggest that the genus *Xanthomonas* diverged from a common ancestor shared with other *Gammaproteobacteria* approximately 100–150 million years ago, coinciding with the diversification of angiosperms [21,22]. Genomic analyses of *X. citri* subsp. *citri* and *X. axonopodis* reveal that adaptation to plant hosts involved the acquisition of virulence determinants, such as T3SS and TALEs, which enabled manipulation of host transcription [23,24]. Positive selection pressure on genes governing host–pathogen interactions, including those encoding lipopolysaccharide (LPS) biosynthesis and effector proteins, further highlights the evolutionary arms race between *Xanthomonas* and its plant hosts [24,25]. These genomic insights underscore the role of ecological niche specialization and adaptive genomic plasticity in shaping the pathogen’s origin.

The discovery of *Xanthomonas* dates to the late 19th century, when bacterial diseases now attributed to this genus were described on several crops, including crucifers, hyacinth, and cotton (*Gossypium* L. spp.) [26,27]. The genus *Xanthomonas* was formally established in the mid-20th century, with *X. campestris* as the type species, following advancements in bacteriological techniques. A pivotal discovery in the 1980s–2000s was the identification of TALEs in *X. oryzae* pv. *oryzae*, which bind host DNA to activate susceptibility genes, revolutionizing understanding of bacterial virulence [28,29]. Subsequent research identified conserved virulence mechanisms, including T3SS and plasmid-mediated gene transfer, as key drivers of pathogenicity across *Xanthomonas* species [30]. These milestones laid the groundwork for molecular studies on host resistance and pathogen evolution.

Historical outbreaks of *Xanthomonas* diseases have reshaped agricultural practices and economies. Citrus canker, caused by *X. citri* subsp. *citri*, emerged in Asia in the early 20th century and spread globally via contaminated plant material, triggering orchard eradication in the Americas [21,31]. Similarly, BLS of corn (*X. vasicola* pv. *vasculorum*) re-emerged in the 2010s in the United States, and exemplifies climate-driven shifts in pathogen distribution [32]. Genomic studies trace the 1980s Florida *X. citri* subsp. *citri* outbreak to a single lineage introduced from Asia [33], while herbarium specimens confirm the pathogen’s presence in 19th-century Indian citrus [21]. These outbreaks underscore the role of human-mediated dispersal and monoculture farming in exacerbating *Xanthomonas* epidemics, necessitating integrated management strategies to mitigate future risks.

### 1.2. Diversity and Taxonomy

The genus *Xanthomonas* exhibits remarkable genetic and ecological diversity, comprising over 30 recognized species and numerous pathovars that infect monocot and dicot hosts across tropical and temperate regions [11]. Recent genomic studies have revealed substantial diversity even within species, driven by HGT, recombination, and host adaptation to diverse plant hosts and environmental niches [34,35]. For example, nonpathogenic *Xanthomonas* strains associated with healthy citrus plants have been identified as novel species, highlighting the ecological versatility of the genus [35]. Comparative genomics has also revealed clade-specific genomic islands and effector repertoires that facilitate host range expansion and environmental resilience [34]. This diversity underscores the evolutionary plasticity of *Xanthomonas*, enabling colonization of both monocot and dicot hosts across tropical and temperate regions [11].

Pathovar classification in *Xanthomonas* historically relied on host specificity and disease symptoms, but molecular tools have exposed limitations in this system. For example, strains classified under *X. arboricola* (infecting walnuts and hazelnuts) and *X. translucens* (causing BLS in cereals) exhibit overlapping host ranges, complicating traditional taxonomic delineation [36,37]. Multilocus sequence typing (MLST) and whole-genome sequencing have resolved ambiguities, leading to the reclassification of strains such as *X. translucens* into novel species *X. cerealis* and *X. graminis* based on phylogenomic criteria [37]. Similarly, *X. arboricola* pathovars were re-evaluated using average nucleotide identity, revealing distinct lineages that correlate with host adaptation [38]. These advancements underscore the need for genomic data to refine pathovar taxonomy and improve diagnostic accuracy.

MLST has emerged as a robust tool for strain differentiation and epidemiological tracking in *Xanthomonas*. Studies on *X. perforans* and *X. euvesicatoria* populations demonstrated that MLST effectively captures geographic and host-associated genetic diversity, resolving strain-specific clusters linked to outbreaks [39,40]. For instance, an MLST scheme targeting *X. citri* pathovars achieved 99.9% accuracy in classifying strains, enabling rapid identification during surveillance [40]. However, MLST’s reliance on conserved housekeeping genes can underestimate diversity in hypervariable regions, such as effector genes governing host specificity [41]. Integrating MLST with whole-genome data enhances resolution, particularly for closely related pathovars like *X. oryzae* pv. *oryzae* and pv. *oryzicola* [41].

Phylogenetic analyses using housekeeping genes (*rpoB*, *gyrB*) and whole-genome data have reshaped understanding of *Xanthomonas* evolution. Early studies identified two major clades separating monocot- and dicot-specific pathogens through 16S–23S rDNA intergenic spacers [42]. Subsequent phylogenomic analyses corroborated this divergence and revealed extensive recombination events, in species such as *X. citri* and *X. campestris*, blurring species boundaries [25,43]. For example, a global phylogeny of *X. citri* subsp. *citri* traced its dispersal from Asia to the Americas, identifying recombination hotspots in virulence-associated regions [25]. These findings highlight the role of HGT in adaptive evolution and the emergence of novel lineages with expanded host ranges.

Taxonogenomic approaches integrating phenotypic, genomic, and phylogenetic data have prompted significant revisions in *Xanthomonas* taxonomy. The reclassification of 20 pathovars into *X. citri* resolved inconsistencies caused by reliance on host-based nomenclature [44]. Similarly, strains formerly grouped under *X. hortorum* were reclassified into *X. hydrangeae*, based on average nucleotide identity (<95%) and core-genome phylogenies [45]. These revisions emphasize the inadequacy of traditional biochemical tests and advocate genome-based criteria such as digital DNA-DNA hybridization and pan-genome analysis for species delineation [37]. Standardizing these methods will enhance taxonomic clarity and facilitate global collaboration in pathogen surveillance.

Host specificity in *Xanthomonas* depends on T3 effector proteins, including TALEs, which interact with plant immune receptors or susceptibility genes [19,46]. For example, *X. euvesicatoria* TALEs target susceptibility genes in pepper, while *X. oryzae* pv. *oryzae* effectors evade rice resistance proteins [47]. Genome-wide studies have identified loci associated with host jumps, such as a *X. perforans* lineage adapting to tomato through mutations in effector *avrXv3* [46]. Conversely, nonhost resistance in *Arabidopsis thaliana* (L.) Heynh. (Arabidopsis) involves recognition of conserved pathogen-associated molecular patterns like flagellin, which are under purifying selection in *Xanthomonas* [19]. Understanding these molecular dynamics informs breeding for durable resistance and targeted biocontrol strategies as well as clarifies evolutionary relationships within the genus.

### 1.3. Xanthomonas Structural and Genomic Characteristics

Recent advances in whole-genome sequencing have significantly enhanced our understanding of *Xanthomonas* evolution, host adaptation, and pathogenicity. High-quality genome assemblies of diverse species, such as *X. hortorum* pv. *pelargonii* and *X. hyacinthi*, have revealed conserved pathogenicity islands and species-specific genomic adaptations [48,49]. For example, the genome of *X. cucurbitae*, the causal agent of bacterial spot in cucurbits, encodes unique virulence factors, including T3SS components and effector proteins, which are absent in non-pathogenic strains [50]. Comparative genomics has also identified HGT events in *X. translucens*, explaining the emergence of novel virulence traits in cereal pathogens [51]. These studies underscore the utility of WGS in tracing outbreaks, identifying diagnostic markers, and predicting virulence mechanisms across *Xanthomonas* pathovars.

The T3SS is a critical virulence determinant in *Xanthomonas*, enabling the delivery of effector proteins into host cells to suppress plant immunity. Functional studies in *X. oryzae* demonstrate that the *MinCDE* cell division system modulates T3SS gene expression and bacterial motility [52]. Structural analyses of the T3SS apparatus in *X. campestris* pv. *vesicatoria* identified HpaB as a chaperone essential for effector translocation, highlighting the complexity of secretion machinery assembly [53]. Additionally, small-molecule inhibitors targeting T3SS activity, such as ortho-coumaric acid and coronatine-like compounds, have been shown to attenuate virulence without affecting bacterial growth, offering promising anti-virulence strategies [54,55]. These findings emphasize the T3SS as both a key virulence hub and a potential target for sustainable disease management.

Plasmids in *Xanthomonas* often carry accessory genes that enhance virulence, host range, and environmental adaptability. For example, the hypervirulent *X. oryzae* pv. *oryzae* strain YNCX harbors six plasmids encoding T3SS effectors and toxin–antitoxin systems, which likely contribute to its aggressive phenotype [56]. Comparative analyses of *X. euvesicatoria* pv. *perforans* strains revealed plasmid-mediated gene reshuffling, including the acquisition of transposons and antibiotic resistance genes, driving pathogen diversification [57]. However, plasmid carriage is not universal within the genus: *X. axonopodis* pv. *citrumelo* lacks detectable plasmids [58], and epidemic plasmid-free lineages of *X. campestris* pv. *campestris* have also been reported [59,60]. The success of these plasmid-free pathovars suggests that key virulence, host-range and stress-adaptation loci can be borne on chromosomal genomic islands rather than plasmids, with functionally similar traits distributed differently across genomes in distinct lineages. These contrasting genomic architectures highlight both the importance of plasmids as mobile genetic elements that facilitate rapid evolution and the capacity of *Xanthomonas* to compensate for plasmid loss through chromosomal integration of virulence determinants.

Effectors are central to *Xanthomonas* pathogenicity, directly manipulating host cellular processes. TALEs in *X. oryzae* activate susceptibility genes in rice (e.g., *OsSWEET14*), while host executor genes in plants trigger resistance by inducing cell death upon TALE recognition [61]. Non-TALE effectors, such as *XopAU* in *X. euvesicatoria*, disrupt host MAP kinase signaling via kinase activity, suppressing immune responses [62]. Structural studies of *AvrBsT* in *X. campestris* pv. *vesicatoria* revealed its role in ubiquitination-mediated immune suppression, illustrating the functional diversity of effector mechanisms [4]. These insights into effector diversity and host targets provide a foundation for engineering resistance genes and developing effector-specific biocontrol agents.

Comparative genomic studies of *Xanthomonas* species have revealed critical insights into their evolutionary adaptation, host specificity, and virulence mechanisms. For example, Ledman et al. [63] demonstrated that *X. translucens* pv. *undulosa* strains isolated from wild grasses and cultivated rice exhibit strain-specific genomic islands encoding T3SS effectors and toxin-antitoxin systems, suggesting niche-specific adaptation. Similarly, Gordon et al. [64] identified three distinct clades within *X. citri* subsp. *citri* strains, with plasmid-borne virulence factors (e.g., *pthA*) driving host-range divergence in citrus canker pathogens. Non-pathogenic *Xanthomonas* species, such as *X. indica*, lack canonical virulence clusters (e.g., T3SS and xanthomonadin biosynthesis) but retain metabolic genes for plant colonization, highlighting evolutionary trade-offs between pathogenicity and symbiosis [65]. These findings underscore the role of HGT and genome plasticity in shaping *Xanthomonas* species pathogenicity, providing targets for resistance breeding and strain-specific diagnostics.

CRISPR-Cas systems in *Xanthomonas* species serve as adaptive immune mechanisms against bacteriophages and mobile genetic elements, while also offering insights into strain diversity and evolution. Hong et al. [66] resolved the structural interaction between CRISPR RNA and anti-CRISPR proteins in *Xanthomonas albilineans*, revealing how phage-derived inhibitors modulate bacterial immunity. Comparative studies of *Xanthomonas citri* subsp. *citri* CRISPR arrays identified hypervariable spacer regions that correlate with geographic origin, enabling high-resolution strain tracking during citrus canker outbreaks [67]. Intriguingly, CRISPR arrays in *X. arboricola* pv. *juglandis* were found to lack functional Cas proteins, suggesting evolutionary degradation in favor of plasmid-mediated defense systems [68]. These observations highlight CRISPR diversity as both a molecular fingerprint for epidemiological studies and a potential tool for phage-based biocontrol strategies against xanthomonad pathogens.

LPS biosynthesis in *Xanthomonas* is critical for membrane integrity, host immune evasion, and virulence. Steffens et al. [69] demonstrated that mutations in LPS O-antigen biosynthesis genes (e.g., *wzt*) disrupt xanthan exopolysaccharide production and biofilm formation in *X. campestris* pv. *campestris*, linking LPS structure to extracellular matrix dynamics. Wang et al. [70] identified the *wxocB* gene in *X. oryzae* pv. *oryzicola* as essential for O-antigen acetylation, with mutants showing impaired motility and reduced activation of rice immune responses. Structural analysis of *X. citri* LPS revealed a conserved lipid A core but variable O-polysaccharide chains that evade plant pattern-triggered immunity (PTI), enabling prolonged survival in the apoplast [71]. Comparative and population genomic studies further show that LPS biosynthesis loci are highly dynamic hotspots of recombination and HGT in xanthomonads infecting rice, sugarcane, citrus and crucifers, leading to rapid gain, loss, and shuffling of O-antigen biosynthetic genes [72,73]. Together with evidence for parallel changes consistent with convergent evolution in pathogens of monocots and dicots [72], these findings suggest strong adaptive selection on LPS structure for immune suppression and niche adaptation. Targeting LPS biosynthesis pathways could thus offer novel strategies for disrupting bacterial adhesion and enhancing host recognition.

## 2. Xanthomonas Major Plant Diseases

### 2.1. Bacterial Leaf Blight of Rice (X. oryzae pv. oryzae)

Recent studies have elucidated the molecular mechanisms underlying the severe virulence of *X. oryzae* pv. *oryzae*, particularly its ability to evade host resistance through genetic mutations. Hypervirulent *X. oryzae* pv. *oryzae* strains, such as C9-3, exhibit expanded effector repertoires, including TALEs that reprogram rice susceptibility genes [74,75]. For instance, African *Xanthomonas oryzae* pv. *oryzae* strains deploy unique TALEs to activate *OsSWEET14*, a sugar transporter critical for bacterial proliferation [75]. Comparative genomic analyses reveal that strain-specific mutations in TALE-binding domains enable pathogen adaptation to resistant rice cultivars, highlighting evolutionary arms races between *X. oryzae* pv. *oryzae* and its host [76]. These findings underscore the role of effector diversity in driving severe blight outbreaks.

Advances in host–pathogen interaction studies have identified novel resistance mechanisms countering *X. oryzae* pv. *oryzae* genetic plasticity. CRISPR-Cas9-mediated editing of *OsSWEET* promoters disrupted TALE-binding sites, conferring broad-spectrum resistance without yield penalties [77]. Similarly, genome-wide association studies pinpointed *Xa10-like* executor genes, which trigger hypersensitive responses upon TALE recognition [78,79]. Transcriptomic analyses further revealed that rice resistance correlates with the induction of calcium signaling suppressors (e.g., *OsCaML2*) and microRNAs like *osa-miR1432*, which fine-tune immune responses [80,81]. These discoveries emphasize the potential of pyramiding multiple resistance alleles to mitigate evolving *X. oryzae* pv. *oryzae* threats.

Classical race analyses, based on infection of differential rice lines carrying single resistance genes, have defined numerous *X. oryzae* pv. *oryzae* races that differ in their effector complement and ability to overcome specific *R* genes such as *Xa4*, *xa5*, *Xa7*, *Xa21*, and *Xa23* [3,15,76,79]. These races fit a gene-for-gene interaction framework in which TAL effectors or other avirulence determinants (e.g., *AvrXa7*, *AvrXa10*, *AvrXa23*) are specifically recognized by matching *NLR* or executor genes (*Xa1/Xa47*, *Xa10*, *XA23*, *Xa27*), triggering effector-triggered immunity (ETI), whereas loss or diversification of the corresponding effectors generates virulent pathotypes [5,28,61,82,83,84]. Population genomic surveys across Asia and Africa show that local *X. oryzae* pv. *oryzae* populations are structured by the *R* gene composition of cultivated varieties, with rapid sweeps of races that overcome widely deployed genes such as *Xa4* or *xa5* [41,76,85]. Understanding this race structure is essential for designing *R* gene pyramids and for predicting the durability of CRISPR-based editing of SWEET promoters, which directly targets TALE-mediated ETS rather than classical ETI [77,86,87,88].

Innovative strategies targeting *X. oryzae* pv. *oryzae* genetic vulnerabilities have emerged. Subtractive genomics identified *folP* and *dnaE*, essential genes for folate biosynthesis and DNA replication, as promising therapeutic targets [89]. Phage cocktails leveraging host range specificity disrupted *X. oryzae* pv. *oryzae* biofilms and suppressed virulence gene expression (*eps* and *rpf* clusters), reducing lesion lengths by 60–80% in field trials [90]. Additionally, riboflavin-mediated photodynamic therapy induced oxidative stress, degrading bacterial membranes and downregulating QS pathways [91]. Such approaches exploit *X. oryzae* pv. *oryzae* metabolic dependencies and regulatory networks to curb infection.

The integration of population genomics and synthetic biology has deepened insights into *X. oryzae* pv. *oryzae* adaptive mutations. Hypervirulent lineages exhibit expanded accessory genomes enriched in transposases and pathogenicity islands, facilitating HGT [76,85]. Ethylicin, a novel quorum-quenching compound, inhibited *rpfF*-dependent diffusible signal factor (DSF) biosynthesis, attenuating virulence in planta [92]. Furthermore, metabolic modeling predicted NADPH-dependent antioxidant pathways as vulnerabilities under host-induced oxidative stress, guiding the design of strain-specific inhibitors [85]. These innovations highlight the importance of targeting genetic and metabolic plasticity to manage *Xanthomonas oryzae* pv. *oryzae* driven blight.

### 2.2. Bacterial Leaf Streak of Rice (X. oryzae pv. oryzicola)

BLS, caused by *X. oryzae* pv. *oryzicola*, is the second major foliar disease of rice after bacterial leaf blight and is increasingly recognized as an emerging constraint in both irrigated and rain-fed production systems. In contrast to *X. oryzae* pv. *oryzae*, which colonizes the xylem, *X. oryzae* pv. *oryzicola* is largely confined to the interveinal mesophyll, producing narrow, translucent, water-soaked streaks that may coalesce into large necrotic areas under conducive conditions. These distinct tissue tropisms underlie the characteristic difference between the blight symptoms of BLB and the linear streaks of BLS, yet the two diseases frequently co-occur in the same fields and can be misdiagnosed [47,93].

Yield losses from BLS are generally lower than from BLB but can still reach ~30% in susceptible cultivars during severe epidemics, particularly in warm, humid lowland environments. Because *X. oryzae* pv. *oryzicola* populations share many core virulence determinants with *X. oryzae* pv. *oryzae* including T3SS, TALEs, QS systems and dynamic LPS loci epidemiological and genomic studies increasingly treat them as a single *X. oryzae* species complex structured by pathovar, pathotype and effector repertoires [11,15,94].

TAL effectors from *X. oryzae* pv. *oryzicola* activate overlapping sets of susceptibility genes with BLB strains, including SWEET sugar transporters and other transcriptional targets, and in some cases are recognized by broad-spectrum resistance loci such as the Carolina Gold Select locus that responds to multiple BLS TAL effectors [93]. This convergence suggests that resistance strategies based on editing SWEET or other S-gene promoters, or stacking executor and NLR genes, can potentially provide dual protection against both BLB and BLS. At the same time, the mesophyll confined lifestyle and distinct environmental sensitivity of BLS (favored by extended leaf wetness and splash dispersal) require tailored management practices and surveillance systems to avoid underestimating its contribution to yield loss.

### 2.3. Citrus Canker (X. citri subsp. citri)

Recent studies have uncovered critical genetic mutations and virulence mechanisms in *X. citri* subsp. *citri* that drive severe infections in citrus bacterial canker. Comparative genomic analyses revealed that *X. citri* subsp. *citri* strains possess a conserved repertoire of pathogenicity factors, including outer membrane proteins and regulators critical for cell membrane integrity and virulence. For example, Wu et al. [95] demonstrated that *X. citri* subsp. *citri* requires a genus-specific outer membrane protein and TolB to coordinate membrane stability and effector secretion, with mutations in these genes significantly attenuating pathogenicity. Similarly, Zhu et al. [96] identified a polyketide cyclase essential for activating the T3SS, highlighting the role of secondary metabolites in modulating bacterial virulence. These findings underscore the genetic complexity underlying *X. citri* subsp. *citri* ability to establish aggressive infections in susceptible citrus hosts.

Novel insights into *Xanthomonas citri* subsp. *citri’s* effector proteins have reshaped understanding of host manipulation. The TALE *PthAW1*, characterized by Teper et al. [97], was shown to hijack host transcription machinery, inducing immune-suppressive pathways that facilitate bacterial proliferation. Additionally, Chen et al. [98] reported that the TALE *PthA4* dynamically reprograms citrus gene expression, particularly upregulating a carbohydrate-binding protein implicated in canker lesion expansion. Cyclic di-GMP signaling, a central regulator of bacterial lifestyle transitions, was also linked to virulence; Shi et al. [99] found that a cyclic di-GMP-binding adaptor protein interacts with a methyltransferase to fine-tune effector delivery and biofilm formation. These studies collectively emphasize the co-evolution of *Xanthomonas citri* subsp. *citri*’s effector arsenal and host-specific immune evasion strategies.

Race structure and host specificity in *X. citri* subsp. *citri* are traditionally described in terms of pathotypes (A, A*, Aw) that differ in host range and aggressiveness on sweet orange, grape, and lime [21,31,64]. These pathotypes are underpinned by variation in TAL effector repertoires, particularly *pthA* alleles that differentially activate the citrus susceptibility gene *CsLOB1* and other host targets [24,98,100,101]. A gene-for-gene-like relationship is evident, in which *PthA* family TAL effectors act as avirulence factors when *CsLOB1* promoters are edited or naturally mutated, abolishing TAL binding and thereby eliminating canker formation [98,101,102]. Phylogeographic studies show that global A-pathotype populations are dominated by a few successful clones whose effector content has been shaped by deployment of partially resistant cultivars and intensive copper use [25,100,103]. Explicitly considering this pathotype structure is critical for evaluating the long-term durability of *CsLOB1* promoter edits and for prioritizing additional susceptibility genes as targets for genome editing in diverse citrus germplasm.

Structural and functional studies have elucidated how *Xanthomonas citri* subsp. *citri* adapts to environmental stress during infection. Barcarolo et al. [104] identified a NADPH quinone reductase critical for mitigating oxidative stress, with knockout mutants exhibiting reduced survival under host-induced reactive oxygen species (ROS). Furthermore, Cabrejos et al. [105] resolved the structure of a superoxide dismutase encoded by a putatively essential gene, revealing its role in neutralizing ROS during apoplastic colonization. Such stress-response mechanisms, coupled with metabolic adaptations like xylose isomerase depletion Alexandrino et al. [106], enable *X. citri* subsp. *citri* to thrive in hostile host environments. These innovations highlight the bacterium’s reliance on redundant genetic pathways to sustain infection under fluctuating host defenses.

Emerging genomic evidence underscores the role of HGT and pathotype diversification in *X. citri* subsp. *citri* evolutionary success. Patané et al. [100] reconstructed the phylogeny of *X. citri* subsp. *citri* pathotypes, linking ancient HGT events to the acquisition of virulence plasmids and host range expansion. Similarly, Zamunér et al. [103] traced the rapid dissemination of a hypervirulent *X. citri* subsp. *citri* lineage across São Paulo’s citrus belt, attributing its dominance to mutations in biofilm-related genes and effector promoters. These genetic adaptations, combined with epigenetic regulation of virulence loci [107], illustrate how *X. citri* subsp. *citri* genomic plasticity drives recurrent epidemics. Together, these advances provide a framework for targeting conserved genetic vulnerabilities in *X. citri* subsp. *citri* to disrupt its pathogenic lifecycle.

### 2.4. Black Rot in Crucifers (X. campestris pv. campestris)

Recent studies have elucidated critical genetic mutations and virulence mechanisms driving severe crucifer black rot infections. ATP-dependent proteases, including *ClpS*, *ClpA*, and *ClpX*, are essential for *X. campestris* pv. *campestris* pathogenicity, enabling stress adaptation and virulence factor regulation. For instance, *clpX* mutants exhibited impaired biofilm formation, reduced oxidative stress tolerance, and attenuated virulence in *Brassica* L. hosts [108]. Similarly, *ClpS*/*ClpA* proteases modulate the degradation of misfolded proteins under host-induced stress, ensuring bacterial survival during infection [109]. These findings highlight the centrality of proteolytic systems in *X. campestris* pv. *campestris* ability to exploit host defenses.

Metabolic adaptations further underpin *X. campestris* pv. *campestris* pathogenicity. The *galU* gene, encoding UDP-glucose pyrophosphorylase, is critical for exopolysaccharide production, motility, and oxidative stress tolerance. *galU* mutants showed reduced EPS synthesis, impaired cell attachment, and diminished virulence in cabbage [110]. Additionally, the FabA-FabB pathway, traditionally linked to fatty acid synthesis, was found to regulate DSF QS in *X. campestris* pv. *campestris*, influencing biofilm formation and systemic invasion [111]. Disruption of *fabA* or *fabB* attenuated DSF production, underscoring metabolic plasticity in *X. campestris* pv. *campestris* virulence strategies.

Novel insights into host–pathogen interactions reveal *X. campestris* pv. *campestris* exploitation of plant transcription factors. In a subset of strains, TAL effectors target *B. oleracea* L. ERF121, a host gene involved in ethylene signaling, to suppress defense responses [112]. However, a recent population-level survey detected canonical TALE genes in <8% of *X. campestris* pv. *campestris* field isolates, indicating that TALE-mediated suppression of ERF121 represents a minority virulence strategy rather than a dominant mechanism at the species level [113]. Concurrently, siderophore-mediated iron acquisition via xanthoferrin is indispensable for bacterial growth in planta, as *xss* mutants (deficient in xanthoferrin synthesis) display reduced survival and virulence in cabbage [114]. These studies emphasize the co-evolution of *X. campestris* pv. *campestris* effector and nutrient-acquisition systems to hijack host resources and evade immunity.

Black rot of crucifers is a classic example of a race-structured pathosystem. At least eleven physiological races of *X. campestris* pv. *campestris* have been described based on their infection profiles on differential *B. oleracea* L. and *B. rapa* L. cultivars, reflecting underlying gene-for-gene relationships between pathogen effectors and host resistance loci [19,115,116]. For example, TAL effectors present in a subset of race 1 and 4 isolates target the *B. oleracea* L. ERF121 transcription factor; natural variation or targeted editing of ERF121 promoter elements alters TAL binding and shifts cultivar-specific resistance [112,113]. Quantitative resistance in *B. napus* L. is further associated with metabolic reprogramming, including enhanced salicylic acid (SA) accumulation and lignification, which restrict systemic spread even when ETI is incomplete [117,118]. Population studies from India and the Caribbean reveal that local race structure is strongly influenced by cultivar deployment and copper-based control, with race 1–like strains and copper-resistant genotypes carrying *copLAB* islands predominating in intensive production systems [115,116,119]. These data highlight the importance of incorporating race surveillance into resistance breeding and integrated management schemes for black rot.

Genetic diversity and HGT contribute to *X. campestris* pv. *campestris* evolving pathogenicity. Indian race 1 strains of *X. campestris* pv. *campestris*, recently reported on *B. juncea*, have been characterized based on their pathogenicity on differential *B.* cultivars and sequence analysis of a fragment of the *hrpF* gene, a component of the *hrp* T3SS, underscoring the genetic and phenotypic diversity present among regional populations [115,116]. Copper resistance genes (*copLAB*) in Trinidadian *X. campestris* pv. *campestris* populations further illustrate adaptive genetic innovations under environmental pressures [119]. Such genomic plasticity, driven by HGT in loci like LPS biosynthesis [73], underscores *X. campestris* pv. *campestris* capacity to acquire traits that exacerbate disease severity in crucifers.

### 2.5. Bacterial Spot of Tomato/Pepper (X. euvesicatoria)

Recent genomic studies highlight the role of genetic recombination and mutation in *X. euvesicatoria* strains driving severe bacterial spot outbreaks in tomato. Comparative analyses by Huang et al. [120] revealed extensive HGT events in tomato-pathogenic strains, enabling rapid adaptation to host defenses. These recombination hotspots often involve virulence-associated loci, such as effector genes in the *avrBs3* family, which suppress plant immune responses. Similarly, Jibrin et al. [121] identified recombination-mediated evolution in Nigerian *X. euvesicatoria* strains, correlating with atypical virulence phenotypes that overcome traditional resistance genes. Such genomic plasticity underscores the pathogen’s ability to evade recognition by tomato immune receptors like *Rx3* [122], leading to unmanaged epidemics in susceptible cultivars.

Bacterial spot of tomato and pepper is organized into well-defined race schemes (T1–T4 in tomato; P0–P10 in pepper) that mirror specific gene-for-gene interactions between *X. euvesicatoria* Avr/TAL effectors and host resistance genes such as *Rx3*, *Rx4*, and the executor gene *Bs3* [122,123,124,125,126]. For instance, AvrBs3-like TAL effectors are recognized by *Bs3* alleles in pepper, triggering hypersensitive cell death (HR) and defining non-permissive races, whereas effector loss or mutation generates races that overcome *Bs3*-mediated resistance [20,125]. Genome-wide association and multilocus variable-number tandem repeat (MLVA) studies show that contemporary field populations are mosaics of recombining lineages whose race composition shifts rapidly in response to resistance gene deployment and copper sprays [46,127,128]. In tomato, quantitative loci for partial resistance to race T4 illustrate how stacking multiple minor-effect genes can buffer against the emergence of new races with altered effector repertoires [46,124]. Explicitly linking these race structures with effector content is therefore a prerequisite for designing durable resistance pyramids and for tailoring phage or biocontrol interventions to local pathogen populations.

Host resistance mechanisms have been a focal point for mitigating severe infections. Genome-wide association studies by Newberry et al. [46] mapped novel quantitative trait loci in tomato germplasm conferring partial resistance to *X. euvesicatoria* race T1. Notably, the *Rx4* gene, identified by Zhang et al. [123], triggers a hypersensitive response upon effector recognition, but its efficacy is strain-specific. Adhikari et al. [124] further demonstrated that resistance to race T4 involves epistatic interactions between multiple quantitative trait loci, suggesting pyramiding strategies may enhance durability. However, the pathogen’s rapid effector repertoire shifts, documented by Potnis et al. [20], challenge single-gene resistance deployment and emphasize the need for dynamic breeding approaches.

Innovative insights into pathogen evolution have emerged from population genomics. Timilsina et al. [127] traced the rise of dominant *X. euvesicatoria* clades in Florida tomato fields to mutations in T3SS regulators, enhancing effector delivery. Concurrently, Liu et al. [129] characterized *SlPUB24*, a tomato E3 ubiquitin ligase that degrades bacterial effectors, but found its activity circumvented by *X. euvesicatoria* strains with mutated *avrXv3* alleles. These findings illustrate an evolutionary arms race, where pathogen mutations in key virulence genes outpace host defenses, exacerbating disease severity in agroecosystems with limited genetic diversity.

In pepper, *X. euvesicatoria*’s effector-driven virulence mechanisms are central to severe bacterial spot. Popov et al. [130] demonstrated that the effector *XopJ4* suppresses SA signaling, enabling systemic infection. Recent work by Drehkopf et al. [131] revealed that T4SS facilitate horizontal transfer of effector genes among strains, accelerating the emergence of hypervirulent lineages in regions like Mexico [132]. These adaptations enable the pathogen to bypass canonical resistance mechanisms, such as the Bs3-mediated hypersensitive response in pepper.

Non-canonical resistance traits in pepper are gaining attention. Bozsó et al. [133] identified a recessive locus in *Capsicum annuum* L. that restricts bacterial colonization without inducing necrosis, suggesting a trade-off between resistance and fitness costs. Subedi et al. [134] further reported that pepper accessions from Southwest Florida exhibit tolerance to multiple *X. euvesicatoria* strains via polygenic mechanisms, though these are strain-specific. Meanwhile, bacteriophage-derived therapies, such as phage BsXeu269p/3 [135], show promise in targeting conserved genomic regions of *X. euvesicatoria*, but field efficacy is limited by the pathogen’s rapid mutation rates in CRISPR-associated genes.

Genomic surveillance has uncovered critical evolutionary trends in pepper-pathogenic *X. euvesicatoria*. Kaur et al. [136] documented the rise of copper-resistant lineages in India, linked to mutations in *copA* and *cusA* genes, which mitigate metal toxicity. Similarly, Vancheva et al. [128] developed a MLVA scheme that revealed Balkan strains have undergone reductive evolution, losing non-essential genes to optimize fitness in pepper hosts. These genomic adaptations, coupled with strain-specific genome rearrangements [137], highlight the pathogen’s capacity to exploit host vulnerabilities and chemical controls, driving recurrent epidemics in intensive production systems (Table 1).

## 3. Molecular Mechanisms of Pathogenesis

### 3.1. Infection Cycle

*Xanthomonas* spp. employ sophisticated molecular strategies to invade host plants, colonize apoplastic spaces, and proliferate within vascular tissues, culminating in devastating diseases. The infection cycle begins with bacterial entry through natural openings like stomata or wounds, a critical step that requires evasion of plant innate immunity. Recent studies reveal that *X. campestris* pv. *campestris* exploits N-acetylglucosamine (*GlcNAc*) during early infection, utilizing a specialized carbohydrate system to bypass plant defenses and establish apoplastic colonies [138]. In rice, *X. oryzae* pv. *oryzae* typically enters via hydathodes and wounds and rapidly colonizes xylem vessels, whereas *X. oryzae* pv. *oryzicola* predominantly enters through stomata, remains largely confined to the mesophyll and interveinal spaces, and spreads laterally within the leaf blade. This difference in tissue tropism underlies the contrasting blight and streak symptomatology of the two major *X. oryzae* rice leaf diseases [47,93]. Similarly, *Xanthomonas citri* leverages wound sites on citrus fruit to initiate infection, with artificial inoculation models demonstrating that mechanical damage facilitates bacterial penetration and systemic spread [139]. Stomatal entry is further modulated by DSF-regulated virulence factors, as shown in *X. campestris*, which suppresses stomatal closure in Arabidopsis to enhance invasion efficiency [140]. Intriguingly, rice cultivars with stomatal mega-papillae exhibit reduced pathogen ingress, highlighting the importance of host structural adaptations in limiting bacterial entry [141]. These findings underscore the dynamic interplay between pathogen virulence mechanisms and plant structural defenses during initial colonization.

Following entry, *Xanthomonas* thrive in the apoplast by deploying enzymes to degrade plant cell walls and suppress immune responses. *X. campestris* pv. *campestris* processes plant N-glycans via a multi-enzyme system, enabling nutrient acquisition and immune evasion [142]. Biofilm formation, mediated by outer membrane porins like *OprB* in *X. citri*, enhances bacterial survival in the apoplastic environment and promotes host tissue maceration [143]. T6SS also plays a critical role: mutations in *Xanthomonas perforans* T6SS core gene *tssM* impair epiphytic survival and reduce virulence, indicating that T6SS-mediated competition is vital for apoplastic persistence [144]. Additionally, TALEs enable *Xanthomonas* to reprogram host gene expression. For example, *X. citri* TALE *PthAW1* activates susceptibility genes in citrus, facilitating bacterial proliferation [145]. These adaptive strategies highlight the pathogen’s ability to manipulate host metabolism and subvert immune signaling during apoplastic colonization.

Vascular invasion represents the final and most destructive phase of *Xanthomonas* pathogenesis. Systemic spread through xylem vessels is driven by enzymatic degradation of vascular barriers and suppression of host defenses. In rice, *Xanthomonas oryzae* pv. *oryzae* exploits weakened stomatal immunity to infiltrate vascular tissues, where it disrupts water transport and induces wilting [146]. The transcription factor *WRKY53* in rice thickens sclerenchyma cell walls via lignin deposition, a defense response that impedes *Xanthomonas* movement; however, virulent strains overcome this barrier through effector-mediated suppression of host resistance pathways [147]. Carbohydrate metabolism remains central to vascular persistence, as *X. citri* subsp. *citri* utilizes *GlcNAc* to fuel biofilm formation and vascular colonization [138]. These insights into *Xanthomonas* vascular tropism emphasize the pathogen’s reliance on both metabolic adaptation and effector-driven immune evasion to achieve systemic infection. Collectively, these studies advance our understanding of the molecular dialogs governing *Xanthomonas* pathogenesis and identify potential targets for disrupting critical infection stages. By contrast, *X. oryzae* pv. *oryzicola* remains largely in the mesophyll, causing extensive chlorotic streaking with less direct disruption of xylem flow, which has implications for both symptom development and the temporal window in which foliar interventions (e.g., copper or biocontrol sprays) are most effective [15,94].

### 3.2. Virulence Factors

#### 3.2.1. Type III Effectors (AvrBs3, Xop Proteins)

Throughout this review we use the term TALEs to refer to the *AvrBs3/PthA* family of type III effectors that share the characteristic central repeat domain with repeat-variable diresidues (RVDs) that determine DNA-binding specificity [28,29]. *AvrBs3* from *X. euvesicatoria* and *PthA* from *X. citri* were the first TAL effectors described and are paradigmatic members of this family, rather than effectors of a distinct type [28]. We therefore avoid the ambiguous phrasing “*AvrBs3* and TAL effectors” used in some earlier literature and instead refer to “TALEs (*AvrBs3*/*PthA* family)” when discussing this class.

The T3SS and its associated effectors are central to the virulence of *Xanthomonas* spp., enabling these pathogens to suppress plant immunity and manipulate host physiology. Recent studies have elucidated critical regulatory and structural components of T3SS assembly. For instance, the *MinCDE* cell division system in *X. oryzae* pv. *oryzae* was found to regulate T3SS gene expression, bacterial motility, and virulence, suggesting a link between cell cycle dynamics and effector deployment [52]. Additionally, the chaperone HpaB in *X. euvesicatoria* governs the translocation of both effector and non-effector proteins by recognizing a conserved N-terminal motif in the regulator HpaA, a process essential for pathogenicity [53,148]. Structural studies further revealed that HrpB4 and HrpB7 in *X. campestris* pv. *vesicatoria* act as scaffolding proteins, facilitating the docking of the T3SS sorting platform to the secretion apparatus, analogous to the role of SctK/YscO proteins in other Gram-negative bacteria [149,150]. These findings underscore the complexity of T3SS regulation and highlight potential targets for disrupting effector delivery.

TALEs (AvrBs3/PthA family) represent a major class of *Xanthomonas* effectors that reprogram host transcription by binding to specific effector-binding elements in promoter regions [28,29]. In *X. oryzae* pv. *oryzae*, TAL effectors such as PthXo1, AvrXa7, and Tal6b activate rice *SWEET* sugar transporter genes or other susceptibility genes, thereby promoting ETS [5,76,86,151]. In *X. citri* subsp. *citri*, PthA alleles activate *CsLOB1* and additional host targets to drive canker formation [98,101,102], while in *X. campestris* pv. *campestris* and *X. euvesicatoria*, conserved TAL effectors target ERF121 in *Brassica* and Bs3 in pepper, respectively, defining classical gene-for-gene relationships [112,125,126]. Truncated TAL effectors can further modulate these interactions by competitively binding to host targets without triggering resistance, thereby dampening executor-gene mediated HR [61,152].

Non-TALE type III effectors, including *AvrBs2*, *AvrRxo1*, *XopN*, *XopQ*, *XopL*, and *AvrXccB*, employ diverse molecular strategies to subvert host defenses. *AvrXccB* from *X. campestris* pv. *campestris* targets putative plant methyltransferases to suppress innate immunity in Arabidopsis, illustrating how effectors disrupt host signaling cascades [153]. Similarly, *XopL* in *X. campestris* pv. *campestris* (*Xcc8004*) is indispensable for virulence, directly interfering with plant immune responses [154], while *AvrRxo1* functions as both a toxin and effector by phosphorylating NAD, depleting cellular pools and compromising immunity [155,156]. Machine-learning approaches have identified additional *X. euvesicatoria* effectors, including novel AvrBs3-like proteins, which exhibit host-specific recognition and contribute to pathogen adaptability [157]. Intriguingly, some effectors, like *XopN* and *AvrBs2* in *X. oryzae* pv. *oryzicola*, exhibit dual roles, enhancing virulence while indirectly promoting host photosynthesis, a strategy that may prolong host viability during infection [158,159]. These studies reveal the functional versatility of effectors and their capacity to fine-tune plant pathogen interactions.

Novel insights into T3SS inhibition have emerged as promising avenues for sustainable disease management. Small-molecule inhibitors, such as 1,3-thiazolidine-2-thione derivatives and ortho-coumaric acid, disrupt T3SS function in *X. oryzae* pv. *oryzae* by downregulating key virulence genes, including hrp (hypersensitive response and pathogenicity) clusters, without impairing bacterial growth [160,161]. Similarly, citrus canker management strategies targeting *X. citri* subsp. *citri* T3SS have identified compounds that reduce biofilm formation and effector secretion, thereby attenuating pathogenicity [55]. Zhou et al. [162] demonstrated that chemical inhibition of T3SS in *X. campestris* not only suppresses disease but also minimizes resistance selection pressure, offering an eco-friendly alternative to traditional bactericides. These approaches exploit the T3SS’s dispensability for bacterial survival in vitro, making it a high-precision target for intervention.

Recent research has also uncovered unexpected roles for T3SS-associated proteins in host manipulation. The accessory protein HrpE in *X. oryzae* pv. *oryzae* enhances plant resistance under non-pathogenic conditions, suggesting that some T3SS components may have evolved dual functions in antagonistic and mutualistic interactions [159]. Furthermore, non-effector proteins like Hpa1 in *X. oryzae* pv. *oryzae*, identified as a T3SS translocator, are critical for pore formation in host membranes, enabling effector injection [163]. Such discoveries challenge the conventional dichotomy between structural and effector proteins, emphasizing the system’s integrated nature. Collectively, these advances deepen our understanding of *Xanthomonas* pathogenicity and inform the development of targeted therapies that disrupt effector deployment while preserving beneficial microbiota.

#### 3.2.2. Toxins and Extracellular Enzymes

Recent studies highlight the critical role of Xanthobaccin-related toxins and extracellular enzymes in *Xanthomonas* pathogenicity. The bifunctional effector AvrRxo1, identified in *X. oryzae*, acts as both a toxin and a virulence factor by disrupting host adenosine triphosphate (ATP) synthesis and triggering programmed cell death, illustrating its dual role in suppressing plant immunity and enhancing bacterial survival [155]. Novel bacteriocins, such as Bcn-B and Bcn-C in *X. perforans*, exhibit protease-like activity and broad-spectrum antibacterial effects, suggesting their involvement in microbial competition alongside host manipulation [164]. These findings underscore toxins as multifunctional tools for niche colonization. Meanwhile, extracellular enzymes, including cellulases, proteases, and xylanases, are indispensable for tissue maceration and nutrient acquisition. For instance, the endoglucanase BglC3 in *X. citri* subsp. *citri* is essential for degrading plant cell walls, with mutants showing significantly reduced virulence [165]. Similarly, *X. campestris* relies on T2SS-secreted proteases and xylanases to breach structural barriers, while amylase activity, regulated by the posttranscriptional modulator RsmA, directly correlates with lesion development in host plants [166,167].

Emerging evidence emphasizes the synergistic interplay between these virulence factors. Ethylicin inhibition studies in *X. oryzae* pv. *oryzicola* revealed that suppressed extracellular enzyme production (e.g., cellulase, protease) disrupts biofilm formation and motility, directly impairing pathogenicity [168]. Furthermore, cellulase mutants in *X. oryzae* exhibit attenuated virulence due to compromised enzymatic synergy; cellulases, xylanases, and pectinases collectively amplify immune elicitation and tissue damage [169,170]. These insights redefine extracellular enzymes not merely as degradative tools but as dynamic regulators of bacterial behavior and host interactions. Together, toxins and enzymes represent actionable targets for disrupting *Xanthomonas* virulence networks, offering novel avenues for sustainable disease management strategies.

#### 3.2.3. Quorum Sensing and Biofilm Regulation

QS in *Xanthomonas* spp. is a pivotal regulatory mechanism coordinating virulence and biofilm formation. The DSF family, including branched-chain fatty acids like BDSF, serves as the primary QS signal. Studies demonstrate that DSF-mediated signaling activates histidine kinase RpfC and its cognate response regulator RpfG, triggering downstream pathways that modulate biofilm maturation, EPS production, and swarming motility [171,172,173]. For instance, *X. campestris* pv. *campestris* relies on RpfC-BDSF binding to regulate virulence gene expression, enabling host tissue invasion and symptom exacerbation [174,175]. Similarly, *X. albilineans* employs DSF to coordinate surface polysaccharide synthesis, critical for leaf attachment and survival [176,177]. These findings underscore the conserved role of DSF-family signals in synchronizing bacterial behavior during infection.

Recent insights reveal dynamic interactions between plant-derived signals and *Xanthomonas* QS systems. Host-produced SA and hydroxycinnamic acids alter cytoplasmic pH, activating the RpfB-dependent turnover of DSF signals in *X. campestris* [178,179]. This pH-sensitive mechanism allows pathogens to adapt their QS activity in response to plant defense metabolites, balancing biofilm dispersal with virulence factor production. For example, *X. oryzae* pv. *oryzae* requires RpfB-mediated signal degradation to optimize virulence during rice infection, highlighting the evolutionary adaptation of QS turnover systems to host microenvironments [180]. Such plasticity underscores QS as a dual-function system, integrating both bacterial population density and host-derived environmental cues.

Novel strategies targeting QS and biofilm regulation offer promising avenues for sustainable disease management. Zingerone-based inhibitors disrupt DSF signaling in *Xanthomonas* spp., reducing biofilm formation and swarming without exerting bactericidal pressure [181]. Additionally, engineered interference with DSF biosynthesis via FabH enzyme inhibition blocks branched-chain fatty acid production, attenuating virulence in *X. citri* [182,183]. These approaches exploit the centrality of QS in virulence, providing alternatives to traditional antibiotics while minimizing resistance risks. Collectively, advances in understanding DSF-family signaling and its crosstalk with host defenses highlight QS as a critical node for innovative, ecology-driven pathogen control (Table 2 and Table 3).

## 4. Host–Pathogen Interactions

### 4.1. Plant Immune Evasion

*Xanthomonas* spp. subvert plant immunity by deploying effector proteins that hijack host susceptibility genes, a process termed ETS. TALEs are central to this strategy, directly binding to promoter regions of S genes to activate their expression. For instance, TALE’s in *X. oryzae* target rice *SWEET* sucrose transporters (e.g., *OsSWEET11a*), which are exploited to facilitate nutrient efflux for bacterial proliferation [86,93]. In the BLS pathosystem, *X. oryzae* pv. *oryzicola* TAL effectors similarly exploit SWEET transporters and additional transcriptional targets, and several of these TAL effectors are recognized by the Carolina Gold Select resistance locus, which responds to multiple, sequence-divergent TAL proteins [93,94]. This illustrates how ETS and ETI can be intertwined even within a single *X. oryzae* species complex. Recent studies reveal that some TALE exhibit dual functionality, simultaneously activating host susceptibility genes while suppressing resistance genes. Xu et al. [151] demonstrated that *Tal6b* in *X. oryzae* targets both *OsSWEET11a* and the resistance gene *Xa27*, illustrating a sophisticated mechanism to balance virulence and immune evasion. These findings underscore the evolutionary adaptability of TALE’s in manipulating host transcriptional machinery.

Non-TALEs also contribute to ETS by disrupting immune signaling or cellular processes. AvrBs2, a conserved effector in *Xanthomonas*, promotes virulence by interfering with abscisic acid signaling, thereby suppressing stomatal immunity in pepper [184]. Similarly, XopL in *X. campestris* targets host proton pump interactors to inhibit SA mediated defenses in Arabidopsis [185]. Intriguingly, certain effectors like XopQ trigger immune responses in non-host plants but are silenced in susceptible hosts through epigenetic modifications, highlighting host-specific adaptation [186]. These studies emphasize the functional diversity of *Xanthomonas* effectors in establishing compatibility.

Emerging insights into ETS highlight the interplay between effector redundancy and host genetic background. For example, Ji et al. [61] showed that truncated TALEs neutralize R-gene-mediated resistance by competing with full-length effectors for host targets. Additionally, CRISPR-based disruption of TALE-binding sites in *SWEET* promoters has emerged as a promising strategy to engineer broad-spectrum resistance [86,94]. However, the rapid co-evolution of effectors and host targets complicates durable resistance, necessitating a deeper understanding of effector diversity and host susceptibility networks.

*Xanthomonas* suppresses PTIthrough effectors that directly inhibit early defense signaling. For instance, *XopP* in *X. oryzae* interferes with the E3 ubiquitin ligase *OsPUB44*, blocking *flg22*-induced MAPK activation and ROS bursts critical for PTI [187]. Similarly, *XopAP* disrupts stomatal immunity by inhibiting vacuolar acidification, thereby preventing abscisic acid induced stomatal closure [188]. Effector redundancy further enhances immune suppression; Popov et al. [130] identified multiple effectors in *X. euvesicatoria* that collectively inhibit *flg22*-triggered callose deposition, a hallmark of PTI. These mechanisms enable *Xanthomonas* to overcome physical and chemical barriers at infection sites.

Effectors also target ETI by disrupting NLR receptor signaling or downstream immune outputs. XopG in *X. oryzae* suppresses ETI-associated ROS bursts by sequestering NADPH oxidases, while XopL inhibits callose deposition in pomegranate to facilitate apoplastic colonization [189,190]. Metabolic manipulation is another strategy: AvrRxo1 phosphorylates NAD to deplete cellular NAD pools, thereby compromising both PTIand ETI [156]. Notably, some effectors exhibit dual roles; XopB suppresses ROS production during PTI but also interferes with SA accumulation, illustrating multifunctional virulence strategies [191].

Recent advances reveal novel mechanisms of immune suppression, such as effector targeting of the exocyst complex, which is essential for vesicle trafficking and defense-related secretion [192]. Additionally, effector redundancy ensures robustness; Huang et al. [193] demonstrated that multiple effectors in *X. campestris* collectively inhibit flg22 responses, ensuring PTI suppression even if individual effectors are neutralized. These insights highlight the complexity of *Xanthomonas* immune evasion and the challenges in developing resistance strategies. Targeting conserved effector hubs, such as RLCKs or exocyst components, may offer broad-spectrum solutions to mitigate pathogen virulence.

### 4.2. Resistance Mechanisms

In the context of plant breeding and pathology, it is important to distinguish between immunity often defined as a complete lack of symptoms and bacterial growth and ‘resistance,’ which refers to the host’s ability to limit pathogen proliferation. While molecular studies often refer to innate immunity (PTI/ETI), in the field, most deployed genes confer varying levels of resistance (from partial to high-level qualitative resistance) rather than absolute immunity. Consequently, the stability of this resistance depends heavily on the specific interaction between host *R* genes and pathogen races. In all four focal pathosystems, resistance has been classically interpreted in terms of Flor’s gene-for-gene model, where qualitative resistance arises when a plant resistance (*R*) gene product recognizes a specific pathogen avirulence (Avr) determinant, most often a TALE or other type III effector [5,19,28]. Differential host sets carrying single *R* genes such as *Xa4*, *xa5*, *Xa7*, or *Xa21* in rice, race-specific resistance loci in *Brassica*, and *Bs* or *Rx* genes in pepper and tomato have been used to define races of *X. oryzae* pv. *oryzae*, *X. campestris* pv. *campestris*, and *X. euvesicatoria*, respectively [3,15,20,115,116,122]. Molecular cloning of both R genes and matching effectors in these systems now allows race structure to be described directly in terms of effector repertoires and *R* gene deployment, providing a mechanistic basis for breeding and for predicting the durability of resistance [5,28,46,82,83]. For BLS, major-effect resistance has been mapped in the American heirloom cultivar Carolina Gold Select, where a locus confers recognition of multiple *X. oryzae* pv. *oryzicola* TAL effectors and resistance to diverse BLS strains [93]. Although this locus is not yet cloned, it provides an important example of TAL-centered resistance operating in a mesophyll-colonizing pathosystem and reinforces the idea that breeding targets for BLB and BLS can be aligned around shared effector biology.

The discovery of *Xa21*, a receptor-like kinase in rice, revolutionized understanding of plant resistance to *X. oryzae* pv. *oryzae*. *Xa21* recognizes pathogen-associated molecular patterns via its extracellular leucine-rich repeat domain, triggering immune responses such as ROS bursts and defense-related gene activation [82]. Recent studies reveal that *Xa21*-mediated resistance involves dynamic phosphorylation events regulated by the phosphatase Paladin, which stabilizes *Xa21* during pathogen attack [194]. Additionally, transcriptional networks downstream of *Xa21*, including *WRKY* transcription factors, prime plants for enhanced pathogen recognition [82,195]. Unlike many resistance genes, *Xa21* confers broad-spectrum resistance without inducing HR, suggesting a unique balance between defense activation and resource conservation.

Executor resistance genes, such as *Xa7* and *XA23*, employ contrasting mechanisms. *Xa7*, a non-NLR executor gene, confers recessive, race-specific resistance by binding TALEs to its promoter, triggering HR only when matching TALEs are present [196]. In contrast, *XA23*, an NLR protein, directly traps TALEs to activate HR, providing broader resistance across *Xanthomonas* strains [83,197]. Both genes highlight the evolutionary trade-off between resistance durability and specificity. For example, Xa7’s durability arises from its reliance on TALE binding to a conserved promoter motif, whereas XA23’s broad efficacy stems from its ability to recognize structurally diverse TALEs [194,198].

Emerging insights into NLR diversification reveal how allelic variation expands resistance spectra. The *Xa1* family, including *Xa1* and *Xa47*, recognizes TALEs via integrated decoy domains, enabling strain-specific resistance [84,152]. Ectopic expression studies demonstrate that non-host plants expressing *XA23* or *Xa27B* gain HR-mediated resistance, suggesting executor genes can be engineered into heterologous systems [197,199]. However, fitness costs associated with constitutive HR, such as reduced growth, underscore the need for precise regulation. Recent work on *Xa47* highlights how *NLRs* synergize with *XA21* to amplify immune outputs, offering strategies for stacking resistance genes [200].

Non-host resistance to *Xanthomonas* often involves PTI and ETI interplay. For example, the QS molecule DSF from *Xanthomonas* activates PTI in non-host plants like Arabidopsis, inducing callose deposition and ROS production [201]. Similarly, the *NLR* Roq1 in tobacco recognizes the *Xanthomonas* effector XopQ, triggering ETI via Ca^2+^ influx and SA/JA signaling [117,202]. These findings emphasize that non-host resistance leverages conserved PTI components while integrating species-specific NLRs to block pathogen effectors.

The *ZAR1* resistosome, a calcium-permeable channel formed by the NLR protein ZAR1, exemplifies how PTI and ETI converge. In Arabidopsis, ZAR1 activates upon detecting *Xanthomonas* effectors, causing Ca^2+^ spikes that amplify immune signaling [203]. This mechanism is conserved in crops; for instance, *B. napus* resistance to *X. campestris* involves metabolic reprogramming linked to SA accumulation and lignin biosynthesis [118]. Notably, the EDS1-PAD4-ADR1 node integrates SA signaling across PTIand ETI, enabling robust immunity even when pathogens suppress individual pathways [186,204].

Metabolic and transcriptional adaptations further underpin non-host resistance. In tomato, the ion channel SlCNGC1/14 suppresses XopQ-induced HR by regulating cytosolic Ca^2+^, balancing defense and cell survival [205]. Similarly, *OsWRKY7* in rice enhances PTI by upregulating *PR* genes, while chlorophyll reductase alleles in cassava indirectly boost resistance by altering redox states [206,207]. These studies highlight that non-host resistance is not merely a passive barrier but a dynamic system integrating multiple layers of defense, offering novel targets for engineering broad-spectrum resistance. Host resistance to *Xanthomonas* involves multiple layers, including PRR-mediated recognition, NLR/executor-gene ETI triggered by type III effectors/TALEs, recessive resistance via altered susceptibility targets (e.g., TFIIAγ5 or SWEET promoters), and quantitative resistance controlled by multiple loci. Table 4 summarizes representative classic and recent examples across these categories.

## 5. Disease Management Strategies

### 5.1. Conventional Approaches

#### 5.1.1. Copper-Based Bactericides and Antibiotics (Limitations: Resistance Development)

Copper-based bactericides have long been a cornerstone for managing *Xanthomonas* diseases, but their efficacy is increasingly undermined by the evolution of resistance mechanisms. In many production systems, antibiotic sprays are not part of standard practice due to legal restrictions and stewardship concerns, so field management relies primarily on integrated disease management combining sanitation, certified pathogen-free planting material, resistant cultivars where available, and non-antibiotic bactericides/biologicals. Studies reveal that *Xanthomonas* species, such as *X. euvesicatoria* pv. *perforans* and *X. citri*, rapidly acquire copper resistance (CuR) via HGT of plasmid-borne *cop* and *cus* operons, which encode efflux pumps and periplasmic copper sequestration proteins [120,136]. Field surveys confirm widespread CuR in tomato and citrus pathogens, with resistance frequencies exceeding 80% in some regions, rendering conventional copper sprays ineffective [211,212]. Novel strategies to circumvent resistance include copper nanoparticles (CuNPs), which exhibit superior antibacterial activity by generating ROS and disrupting cell membranes in *X. campestris* pv. *vesicatoria* [213]. Hybrid nanomaterials, such as magnesium-copper composites, further enhance bactericidal effects by combining ion release with physical membrane damage, even against CuR strains [214]. Additionally, small molecules like piperidine and pyrrolidine disrupt biofilm formation and suppress virulence genes in CuR *X. perforans*, offering synergistic potential with copper-based treatments [215]. However, the sustainability of these innovations remains uncertain, as *Xanthomonas* populations exhibit adaptive plasticity through mutation and recombination, necessitating integrated approaches to delay resistance [136].

Antibiotics, particularly streptomycin and oxytetracycline, have historically been used against some bacterial plant diseases, but their utility for *Xanthomonas* management is increasingly limited by (i) rapid resistance evolution and (ii) regulatory restrictions or outright prohibitions in many countries under antimicrobial stewardship frameworks. Where antibiotic use is still permitted, it is typically highly regulated, often restricted to specific crops and situations, and should be considered a last-resort intervention rather than a routine management tool. Resistance in xanthomonads can arise via chromosomal mutations (e.g., rpsL-associated streptomycin resistance) and horizontal acquisition of resistance determinants (e.g., strAB, tet genes), and selection pressure can increase when antibiotics are used repeatedly or prophylactically. Accordingly, contemporary best practice emphasizes minimizing antibiotic reliance by prioritizing clean planting material/seed health, sanitation, copper alternatives where permitted, host resistance, and sustainable biological or anti-virulence approaches (biocontrol consortia, bacteriophages, quorum-quenching molecules, and nanomaterial-enabled antimicrobials), together with resistance monitoring and local compliance with national regulations.

Antibiotics, particularly streptomycin and oxytetracycline, have also faced severe resistance challenges in *Xanthomonas* management. Genetic analyses of *X. arboricola* pv. *pruni* and *X. oryzae* pv. *oryzae* reveal that resistance arises via chromosomal mutations (e.g., *rpsL* mutations conferring streptomycin resistance) and horizontal acquisition of *strAB* or *tetC* genes, which encode detoxifying enzymes [216,217]. Alarmingly, resistance frequencies in field strains now exceed 60% in regions with heavy antibiotic use, driven by selection pressure and mobile genetic elements [218,219]. Metabolic interventions, such as exogenous alanine supplementation, have shown promise in reversing resistance to aminoglycosides by restoring ATP synthesis and membrane permeability in *X. oryzae* [219]. Similarly, structural insights into antibiotic-binding proteins, like AlbA in *X. albilineans*, inform the design of albicidin analogs that evade resistance mechanisms [220]. Nevertheless, regulatory restrictions and environmental concerns limit antibiotic deployment, prompting exploration of alternatives such as phage therapy and resistance-breaking nanomaterials [214,221]. Collectively, these studies underscore the urgent need for resistance monitoring, rotation of chemistries, and combinatorial therapies to prolong the utility of conventional bactericides and antibiotics in *Xanthomonas* disease management.

#### 5.1.2. Sanitation and Quarantine Measures

Sanitation and quarantine measures remain foundational to mitigating the spread of *Xanthomonas* spp., particularly in preventing pathogen introduction and reducing inoculum reservoirs. Recent studies underscore the efficacy of thermal pruning and debris removal in disrupting bacterial survival. For instance, Morvan et al. [222] demonstrated that thermal pruning of wild blueberries reduced pathogen loads in root-associated microbiota, highlighting its dual role in sanitation and microbiome health. Similarly, integrated sanitation practices, such as removing infected plant debris and disinfecting tools, have proven critical in orchard systems to limit *Xanthomonas* persistence [223]. Chlorine-based products (e.g., sodium hypochlorite) and other sanitizers are useful to significantly reduce bacterial viability in postharvest settings, with Stahr et al. [224] reporting a 90% reduction in *Ceratocystis fimbriata* contamination on sweetpotato surfaces a finding broadly applicable to *Xanthomonas* management. These strategies are further enhanced by crop rotation and field sanitation, which disrupt pathogen lifecycles and reduce soilborne inoculum [225].

Quarantine measures are equally vital for containing *Xanthomonas* outbreaks, particularly in global agricultural trade. Advanced molecular diagnostics, such as CRISPR-Cas12a assays, enable rapid detection of latent infections in seeds and planting materials, ensuring compliance with phytosanitary regulations [226]. Jung et al. [227] emphasized the importance of biosecurity protocols in poultry systems, which parallel plant-pathogen management by restricting contaminated equipment and enforcing pathogen-free zones. Additionally, Kavhiza et al. [228] validated the use of commercial DNA extraction kits for sensitive detection of *Xanthomonas euvesicatoria* in onion seeds, reinforcing the need for certified disease-free stocks. Together, these studies advocate for a *“clean start*, *clean finish”* approach, combining rigorous sanitation with regulatory quarantine to curb both local and transnational transmission of *Xanthomonas* pathogens.

### 5.2. Sustainable Means for the Management

#### 5.2.1. Biocontrol Agents

Recent advances in biocontrol research have demonstrated the efficacy of *Bacillus* and *Pseudomonas* spp. in suppressing *Xanthomonas* infections through direct antagonism and systemic plant protection. *Bacillus amyloliquefaciens* FZB42 produces antimicrobial metabolites, including difficidin and bacilysin, which exhibit potent inhibitory activity against *X. oryzae* by disrupting bacterial membrane integrity and metabolic pathways [229]. Similarly, *Pseudomonas aeruginosa* BRp3 secretes secondary metabolites such as phenazines and cyclic lipopeptides, which reduce *Xanthomonas*-induced leaf blight severity in rice by up to 70% while enhancing plant growth through nitrogen fixation and phosphate solubilization [230]. Field trials with *Bacillus velezensis* strains have shown a 50–60% reduction in pepper bacterial spot (*X. euvesicatoria*) incidence, attributed to biofilm formation and competitive exclusion of pathogens [231]. Furthermore, *Paenibacillus polymyxa* Sx3 suppresses *X. oryzae* pv. *oryzae* by inducing ROS-scavenging enzymes in rice, thereby mitigating oxidative stress and improving yield under disease pressure [232]. These studies highlight the dual functionality of biocontrol agents in pathogen suppression and plant growth promotion, though their efficacy depends on rhizosphere colonization efficiency and compatibility with host genotypes [233,234].

#### 5.2.2. Induced Systemic Resistance (ISR) Elicitors

ISR elicitors offer a proactive strategy to prime plant immune responses against *Xanthomonas* pathogens by modulating defense-related pathways. Streptomyces spp., such as *Streptomyces shenzhenesis* TKSC3, activate SA and jasmonic acid dependent signaling in rice, upregulating pathogenesis-related (PR) genes (e.g., *PR1*, *PR10*) and reducing BLS severity by 65% [235]. Similarly, chumacin-1 and chumacin-2, novel QS inhibitors derived from *Pseudomonas aeruginosa* CGK-KS-1, disrupt *Xanthomonas* communication systems, suppressing virulence factor production (e.g., EPS, cellulases) and biofilm formation [236]. Combinatorial applications of ISR-inducing rhizobacteria, such as *Pseudomonas fluorescens* and *Bacillus subtilis* consortia, have shown synergistic effects, enhancing lignin deposition in vascular tissues and reducing *Xanthomonas* translocation in tomato by 80% [234]. These findings underscore the potential of ISR elicitors to reduce chemical pesticide reliance, though their effectiveness varies with environmental conditions and requires precise timing to align with pathogen infection cycles.

#### 5.2.3. CRISPR-Based Genome Editing for Disease Resistance

CRISPR-Cas9 technology has revolutionized the development of *Xanthomonas*-resistant crops by enabling precise editing of susceptibility (S) genes or pathogen virulence targets. In rice, knockout of the promoter region of *SWEET14*—a sugar transporter hijacked by *Xanthomonas oryzae* pv. *oryzae* confers broad-spectrum resistance to 95% of *Xanthomonas oryzae* pv. *oryzae* strains without compromising yield [87]. Because many *X. oryzae* pv. *oryzicola* TAL effectors also target SWEET promoters, these edited alleles often reduce susceptibility to BLS as well, providing dual protection against both major *X. oryzae* leaf diseases with a single genome-edited locus [86,87,88]. However, given the diversity of pathogen races, a prerequisite for this method is that the edited variants must be tested for efficacy against a broad set of local pathogen races and types to ensure the resistance is durable. Similarly, editing the *CsLOB1* promoter in citrus disrupts *Xanthomonas citri* subsp. *citri* effector binding, reducing canker lesion formation by 90% in field trials [102]. Emerging tools like CRISPR-based diagnostic kits enable rapid detection of *Xanthomonas* strains, facilitating timely deployment of resistant cultivars [237]. While regulatory and public acceptance challenges persist, CRISPR-edited crops represent a scalable, environmentally sustainable solution to mitigate *Xanthomonas*-driven yield losses, particularly in staple crops vulnerable to evolving pathogen strains.

Taken together, these approaches illustrate how mechanistic insight into race structure and effector repertoires in *X. oryzae* pv. *oryzae*, *X. citri* subsp. *citri*, *X. campestris* pv. *campestris*, and *X. euvesicatoria* can be translated into sustainable management. For example, CRISPR editing of *SWEET* or *CsLOB1* promoters directly blocks TALE-mediated ETS, while biocontrol consortia and DSF interfering molecules reduce pathogen populations and selection pressure on R genes [86,87,101,102,229,234,238]. Phage cocktails or nanoparticle formulations can then be deployed strategically against local races identified through effector-based genotyping, complementing rather than replacing resistant cultivars [90,214,239,240]. This integration of host resistance, effector-targeted interventions, and ecological tools provides a concrete framework for sustainable *Xanthomonas* management in the four focal pathosystems (Table 5).

## 6. Climate Change Impacts

### 6.1. Changing Disease Dynamics

#### 6.1.1. Temperature/Humidity Effects on Pathogen Spread

Climate-driven shifts in temperature and humidity are reshaping the epidemiology of *Xanthomonas* diseases across agricultural systems. Research demonstrates that warmer temperatures and prolonged humidity significantly enhance pathogen survival, reproduction, and dissemination. For instance, Kilwenge et al. [242] identified a direct correlation between rising temperatures and Banana *Xanthomonas* Wilt (BXW) incidence in Rwanda, where increased moisture from erratic rainfall extended bacterial persistence on plant surfaces. Similarly, Moragrega et al. [243] established critical thresholds for walnut blight (*X. arboricola* pv. *juglandis*), with infection rates peaking at 18–25 °C and leaf wetness durations exceeding 12 h. Such conditions facilitate biofilm formation and stomatal infiltration, as observed in *X. citri* [244]. Furthermore, Abrahamian et al. [245] revealed that overhead irrigation in tomato nurseries a proxy for high humidity amplified *X. perforans* transmission by 40%, underscoring the role of water aerosols in pathogen dispersal. Collectively, these studies suggest that climate change will exacerbate *Xanthomonas* outbreaks in two key ways: (1) expanding geographic ranges into previously cooler regions and (2) intensifying epidemic severity in endemic zones through extended favorable periods for bacterial growth. Predictive models, such as the climate-risk framework for BXW [242], highlight the urgent need for adaptive irrigation practices and humidity-controlled cultivation to disrupt infection cycles.

#### 6.1.2. Emergence of New Strains/Pathovars

Climate stressors are driving the evolution of genetically distinct *Xanthomonas* strains with enhanced virulence and environmental adaptability. Genomic analyses have uncovered climate-linked diversification patterns, such as the emergence of aggressive *X. arboricola* clones adapted to warmer climates [246]. These epidemic strains exhibit upregulated heat-shock proteins and effector genes, enabling survival under thermal stress. In *X. citri*, Bansal et al. [247] documented recombination events between phylogenetically divergent pathovars, likely accelerated by host shifts under climate-induced ecological pressures. Similarly, Kaluzna et al. [248] revealed climate-associated genomic divergence between *X. arboricola* pv. *juglandis* (walnut) and pv. *corylina* (hazelnut), with the former evolving thicker biofilms to withstand arid conditions. Notably, copper-resistant *X. euvesicatoria* pv. *perforans* strains have proliferated in tomato fields, with resistance genes linked to horizontal transfer under climate-stressed environments [136]. Such adaptations not only enhance pathogen resilience but also compromise conventional management strategies, as evidenced by the failure of copper-based treatments in India [136]. These findings underscore a critical feedback loop: climatic extremes select for fitter pathogen genotypes, which in turn drive more severe epidemics. Addressing this challenge requires genomic surveillance to track strain evolution and the development of climate-resilient biocontrol agents tailored to emerging pathovars.

### 6.2. Xanthomonas Adaptation Challenges in Changing Climates

#### 6.2.1. Xanthomonas Reduced Chemical Efficacy Under Extreme Weather

Emerging research highlights the growing challenge of chemical control efficacy against *Xanthomonas* spp. under extreme weather conditions. Elevated temperatures and prolonged humidity have been shown to accelerate bacterial adaptation, reducing the effectiveness of traditional antimicrobial agents. For instance, studies on copper-based treatments for *X. arboricola* pv. *pruni* in walnut orchards revealed increased copper tolerance in bacterial populations exposed to heat stress, correlating with overexpression of metal efflux pumps and biofilm formation [249]. Similarly, nanoparticle-based biopesticides, such as carvacrol nanoemulsions, exhibited diminished bactericidal activity against *X. axonopodis* pv. *cyamopsidis* under drought-like conditions due to altered bacterial membrane permeability and enhanced stress-response pathways [250]. Extreme weather also disrupts host–pathogen dynamics: high temperatures compromised the efficacy of rice resistance gene *Xa7* against *X. oryzae* pv. *oryzae*, as heat stress suppressed host defense signaling while promoting bacterial effector protein expression [251]. Furthermore, accelerated mutation rates in *X. perforans* during seasonal heatwaves led to rapid evolution of streptomycin resistance, underscoring the interplay between climatic stressors and adaptive mutagenesis [252]. These findings emphasize the need for climate-resilient chemical formulations, such as temperature-stable nanocarriers, and integrated strategies combining antimicrobials with host resistance induction to mitigate reduced efficacy.

#### 6.2.2. Xanthomonas Geographic Host Range Expansion

Climate change is driving the geographic expansion of *Xanthomonas* pathogens into previously unsuitable regions, facilitated by shifting temperature and precipitation patterns. Predictive modeling of BXW (*X. campestris* pv. *musacearum*) in Rwanda projected a 15–30% increase in disease incidence by 2050, with warmer temperatures extending the pathogen’s viability in high-altitude regions previously limited by cooler climates [242]. Similarly, *X. oryzae* pv. *oryzae*, the causal agent of rice bacterial blight, is predicted to colonize new agroecological zones in South Asia and sub-Saharan Africa as rising minimum temperatures (>20 °C) and erratic rainfall create favorable conditions for epiphytic survival and transmission [253]. Comparable expansion is expected for BLS, as *X. oryzae* pv. *oryzicola* thrives under warm, humid conditions and often co-occurs with *X. oryzae* pv. *oryzae* in rain-fed lowland rice. Under future climate scenarios, co-epidemics of BLB and BLS may become more frequent, complicating diagnosis and resistance deployment strategies [15,47]. Host range expansion is further enabled by genetic adaptations: comparative genomics of *X. translucens* pathovars identified HGT events involving *tal* effector genes, allowing infection of novel grass hosts in drought-stressed environments [254]. Additionally, *X. citri* pv. *viticola* has exploited warming winters in Mediterranean climates to infect previously resistant grapevine cultivars, with genomic evidence pointing to cold-shock protein upregulation enhancing low-temperature resilience [255]. These trends underscore the urgency of global surveillance networks and climate-smart breeding programs to preempt outbreaks in newly vulnerable regions.

## 7. Future Perspectives

*Xanthomonas* spp. are now among the best characterized bacterial plant pathogens in terms of effector biology, race structure, and population genomics, yet major gaps remain in translating this mechanistic knowledge to durable, field level control. Future research on *Xanthomonas* should move beyond single-gene or single-pathosystem case studies toward comparative, systems-level frameworks that integrate genomics, ecology, climate, and management across crops [2,11]. The four focal pathosystems in this review, bacterial leaf blight and leaf streak of rice, citrus canker, black rot of brassicas, and bacterial spot of tomato and pepper provide a strong foundation for such a framework because they jointly span monocot and dicot hosts, annual and perennial systems, and both temperate and tropical production environments.

On the research side, a first priority is to more explicitly connect effector repertoires, race structure, and epidemiology. Although TAL effectors and other type III effectors have been characterized in many *Xanthomonas* populations, routine, high-throughput effector profiling is still not part of most breeding and surveillance programs [15,27,29]. Future work should (i) standardize effector-based genotyping schemes across laboratories and regions, (ii) link effector and race data to field performance of resistance genes and edited susceptibility alleles, and (iii) model how deployment of specific R-gene or S-gene combinations shapes the evolutionary trajectories of local *Xanthomonas* populations. Across pathosystems, coordinated sampling of both pathogenic and nonpathogenic *Xanthomonas* lineages, including endophytes and epiphytes, will also be critical for understanding how genomic plasticity, HGT, and lifestyle shifts drive the emergence of new diseases and host jumps [6,35,65].

A second research priority is to embed *Xanthomonas* biology into climate change and landscape level contexts. Existing studies already show that temperature, humidity, and rainfall strongly affect *Xanthomonas* survival and epidemic dynamics, and that climate change is likely to expand the geographic range of key diseases such as bacterial leaf blight, BXW, and walnut blight [242,243,253]. Going forward, multi-scale models that combine climate projections, crop distribution, and pathogen genomic data will be needed to (i) identify future hotspots where BLB/BLS co-epidemics or citrus canker outbreaks are likely to intensify, (ii) anticipate where copper-resistant or hypervirulent lineages are most likely to establish, and (iii) prioritize surveillance and breeding targets accordingly [21,25,136]. *Xanthomonas*, with its well-resolved phylogenies and race schemes, is an ideal model to develop such climate-smart risk-mapping pipelines.

On the management side, future work should focus less on evaluating individual tools in isolation and more on designing integrated, effector-informed management packages tailored to specific production systems. For rice, this means combining (i) edited SWEET and other susceptibility gene promoters that provide dual protection against BLB and BLS, (ii) the pyramiding of executor and NLR gene stacks such as *Xa21*, *Xa7*, *Xa1/Xa47*, and *XA23*, and (iii) agronomic practices, biocontrol agents, and anti-virulence chemistries that reduce inoculum pressure and slow effector evolution [15,77,86,87,92]. For citrus, integrating CsLOB1 promoter edits with pathotype-specific surveillance, optimized copper or nanoparticle formulations, and biologicals will be crucial to manage both classical canker and newly emerging lineages [21,100,102]. In brassicas and solanaceous crops, race-specific resistance, quantitative resistance loci, phage cocktails, and DSF or T3SS-targeting compounds must be deployed within robust sanitation and seed/seedling health programs to maintain efficacy in the face of frequent recombination and copper resistance [20,117,127,136].

One critical bottleneck often overlooked is the establishment of a highly effective screening system. This is a cardinal point for two reasons: identifying the best plants from sophisticated genetic material and screening germplasm for new sources of resistance. Since different isolates of the same species can show large differences in aggressiveness, resistance rankings can vary significantly based on the inoculum used. Future protocols must move beyond single-isolate inoculations to include multi-strain mixtures and geographically diverse isolates. This ensures that the amount of resistance is characterized against the full spectrum of pathogen virulence.

Emerging technologies such as CRISPR-based genome editing, phage therapy, nanomaterials, and quorum-quenching approaches should be viewed not as stand-alone silver bullets but as components of such integrated strategies. For example, CRISPR editing of *SWEET* and *CsLOB1* promoters has already demonstrated that susceptibility gene engineering can deliver high levels of resistance with limited yield penalty [86,87,102]. Future work should extend this concept to additional susceptibility targets identified through population genomics and effector biology, while systematically monitoring potential fitness and ecological trade-offs. Similarly, phage cocktails and nanoparticle formulations will need to be designed with attention to race structure, local regulations, environmental fate, and compatibility with biocontrol agents and chemical inputs [90,214,239,240]. Integrating real-time diagnostics, including CRISPR-based field assays, into these management packages could enable more responsive, spatio-temporally targeted interventions [226,237].

Finally, implementation of science and socio-economic considerations must become central to *Xanthomonas* research agendas. Many of the most severe *Xanthomonas* diseases including bacterial leaf blight and leaf streak of rice, cassava bacterial blight, BXW, and citrus canker disproportionately affect smallholder farmers in low- and middle-income countries [7,8,11]. Future work should therefore (i) co-design management strategies with growers and extension systems, (ii) evaluate the affordability, accessibility, and gender and equity implications of new technologies such as edited cultivars or nanomaterials, and (iii) invest in regional networks for effector and race monitoring, seed and seedling certification, and decision-support tools that integrate weather, disease risk, and resistance deployment. *Xanthomonas* pathosystems provide a powerful testbed for building such translational pipelines, because they combine well-defined molecular targets with clear and urgent food-security impacts.

In summary, the next phase of *Xanthomonas* research and management should prioritize (i) effector-aware, climate-informed surveillance, (ii) rational design and monitoring of resistance and anti-virulence strategies at the population level, and (iii) equitable, context-appropriate implementation in diverse production systems. Achieving these goals will require sustained collaboration among plant pathologists, breeders, microbiologists, modelers, social scientists, and policymakers. If these interdisciplinary efforts can be realized, *Xanthomonas* spp. may shift from being emblematic of emerging bacterial threats to serving as a model for how to build resilient, sustainable management systems for plant bacterial diseases in a changing climate.

## 8. Conclusions

*Xanthomonas* spp. represent a persistent and evolving threat to global agriculture, driven by their genetic adaptability, effector-mediated virulence, and capacity to exploit environmental and host vulnerabilities. In this review we have brought together race structure, effector biology, and sustainable management options for four model pathosystems: the two major rice leaf diseases, bacterial leaf blight and BLS, citrus canker, black rot of brassicas, and bacterial spot of tomato and pepper. By viewing these diseases through a shared lens of TAL effector repertoires, susceptibility genes and quantitative resistance loci, we identify common principles that can guide effector-informed, climate-resilient management across diverse crops.

Conventional management strategies, including copper-based bactericides and antibiotics, are increasingly undermined by resistance development and by reduced efficacy under extreme weather, highlighting the necessity for sustainable alternatives. Because antibiotic use in crop protection is restricted or prohibited in many countries and carries substantial AMR stewardship concerns, antibiotics should not be viewed as a sustainable cornerstone for *Xanthomonas* control, reinforcing the need to scale non-antibiotic integrated strategies. Advances in CRISPR-based genome editing now enable precise targeting of susceptibility genes such as rice SWEET transporters and citrus CsLOB1, often conferring protection against both bacterial leaf blight and leaf streak with minimal yield penalty. In parallel, biocontrol agents, quorum-quenching molecules and nanoparticle formulations offer eco-friendly tools to reduce pathogen load and selection pressure on host resistance.

A key conclusion from our comparative approach is that bacterial leaf blight and BLS of rice should be treated as a single *X. oryzae* disease complex in surveillance, breeding and management, given their shared virulence mechanisms and overlapping resistance targets but distinct tissue tropisms and climate sensitivities. More broadly, integrating population genomics, race monitoring, and effector-based diagnostics with host resistance pyramids, phage biocontrol and nanotechnology provides a concrete framework for durable control of *Xanthomonas* diseases.

Future efforts must prioritize combinatorial strategies, for example, CRISPR-edited S-gene promoters deployed together with executor and NLR gene stacks, DSF- or T3SS-targeting chemistries, and climate-smart surveillance systems to pre-empt outbreaks and slow resistance evolution. Collaborative, multidisciplinary research that connects plant pathology, genomics, synthetic biology and socio-economic analysis will be essential to translate these innovations into field-applicable solutions. Addressing the complex challenges posed by *Xanthomonas* requires a holistic framework that combines traditional practices with cutting-edge technologies, emphasizing resilience, equity and ecological balance in global crop protection strategies.

## Figures and Tables

**Table 1:** Plant diseases caused by *Xanthomonas* species treated in this study.

Disease	Pathogen	Host Plants	Key Symptoms	Economic Impact	References
**Bacterial leaf blight**	*X. oryzae* pv. *oryzae*	*Oryza sativa* (Rice)	Linear water-soaked lesions, wilting, yield loss	Up to 75% yield loss in endemic regions; threatens food security in Asia	[3]
**BLS**	*X. oryzae* pv. *oryzicola*	*Oryza sativa* (Rice)	Narrow, translucent water-soaked streaks between veins that become yellow–brown and necrotic	Yield losses up to ~30% in susceptible cultivars; frequently co-occurs with bacterial leaf blight and complicates diagnosis and resistance breeding	[15,47,93,94]
**Citrus canker**	*X. citri* subsp. *citri*	*Citrus* spp. (Citrus)	Raised corky lesions, defoliation, premature fruit drop	Extensive orchard destruction; costly eradication programs in the Americas	[7,21]
**Black rot**	*X. campestris* pv. *campestris*	*B. oleracea* (Cabbage, Broccoli)	V-shaped chlorosis, vascular blackening	Reduces crop quality; significant losses in vegetable production	[108,116]
**Bacterial spot**	*X. euvesicatoria* pv. *perforans*	*Solanum lycopersicum* (Tomato), *Capsicum annuum* (Pepper)	Necrotic spots, defoliation, premature leaf drop	Reduces marketability; global losses in solanaceous crops	[20,127]

**Table 2:** Virulence Mechanisms of *Xanthomonas* spp.

Mechanism	Function	Examples	References
**T3SS**	Delivers effector proteins into host cells	HpaB (chaperone), HrpB4, HrpB7 (structural)	[52,53]
**TAL effectors**	Reprogram host gene expression by binding DNA promoters	AvrBs3, PthA4 (activates *OsSWEET* genes)	[28,98]
**Extracellular enzymes**	Degrade plant cell walls for nutrient acquisition	Cellulases, proteases, xylanases	[110,170]
**DSF QS**	Regulates biofilm formation and virulence	RpfC/RpfG system	[172,173]
**LPS**	Evade plant immunity; maintain membrane integrity	O-antigen acetylation (*wxocB* gene)	[70,71]

**Table 3:** Comparative summary of similarities and dissimilarities in molecular mechanisms across major *Xanthomonas* pathosystems.

Mechanism Category	Similarities	Dissimilarities	References
**Infection cycle and tissue tropism**	**Entry:** All species utilize natural openings (stomata, hydathodes) or mechanical wounds.	**Vascular vs. mesophyll:** *X. oryzae* pv. *oryzae* and *X. campestris* pv. *campestris* invade xylem vessels causing systemic wilting; *X. oryzae* pv. *oryzicola*, *X. citri*, and *X. euvesicatoria* remain in the mesophyll/parenchyma causing streaks or spots.	[47,93,138]
	**Establishment:** Initial apoplastic phase involving immune suppression and nutrient acquisition is universal.	**Entry specificity:** *X. oryzae* pv. *oryzicola* preferentially enters via stomata; *X. oryzae* pv. *oryzae* via hydathodes/wounds.	
**Toxins and extracellular enzymes**	**Enzymes:** Widespread secretion of Cell Wall Degrading Enzymes (cellulases, xylanases, proteases) via T2SS to macerate tissue.	**Specific toxins:** *X. oryzae* deploys AvrRxo1, a bifunctional toxin that phosphorylates NAD and depletes ATP.	[155,164,165]
	**Function:** Dual role in nutrient acquisition and virulence.	**Bacteriocins:** *X. perforans* produces Bcn-B and Bcn-C for microbial competition.	
		**Bacteriocins:** *X. perforans* produces Bcn-B and Bcn-C for microbial competition.	
**QS and biofilm**	**DSF system:** The RpfC/RpfG two-component system and DSF are conserved regulators of virulence and motility.	**Signal turnover:** *X. campestris* and *X. oryzae* exhibit RpfB-mediated signal turnover triggered by host SA or pH changes.	[143,172,178]
		**Biofilm adaptation:** *X. citri* utilizes OprB porins and c-di-GMP to stabilize biofilm on fruit surfaces; *X. arboricola* forms thicker biofilms for drought tolerance.	

**Table 4:** Host Resistance Mechanisms Against *Xanthomonas* Infections.

Resistance Gene/Locus	Host Plant	Pathogen Targeted	Mechanism	Effectiveness	References
***Xa21***	Rice	*X. oryzae* pv. *oryzae*	Receptor kinase recognizing RaxX sulfopeptide	Durable resistance in field conditions	[194,208]
***Xa4***	Rice	*X. oryzae* pv. *oryzae*	Broad, durable resistance associated with strengthened basal defenses (classic R gene)	Reduces vascular colonization and symptom severity	[3,15]
***xa5* (TFIIAγ5 allele)**	Rice	*X. oryzae* pv. *oryzae*	Recessive resistance; reduces TALE-dependent transcriptional activation of susceptibility	Broad/partial resistance across races; widely deployed	[3,15]
***xa13*/*OsSWEET11* promoter variant (*Os8N3*)**	Rice	*X. oryzae* pv. *oryzae*	Recessive resistance via loss of TALE binding to SWEET promoter (S-gene/ETS disruption)	Strong reduction of blight when matching TALE cannot induce SWEET	[3,15]
***Xa1*/*Xa47***	Rice	*X. oryzae* pv. *oryzae*	NLR-mediated recognition of TALE activity (ETI); may be suppressed by interfering TALEs	Broad-spectrum resistance in some genetic backgrounds	[84,152]
***Xa23* (executor R gene)**	Rice	*X. oryzae* pv. *oryzae*	TALE-inducible executor gene triggers HR (executor/ETI)	Strong, often broad resistance where inducing TALE is present	[61,83,198]
***Xa27*/*Xa10*-*like* executors**	Rice	*X. oryzae* pv. *oryzae*	TALE-inducible executor genes trigger localized cell death (executor/ETI)	Race-specific but high-effect resistance	[61,78]
**Carolina Gold BLS** **resistance locus**	Rice	*X. oryzae* pv. *oryzicola*	Broad, TAL effector dependent ETI; recognition of multiple BLS TAL effectors	Broad-spectrum resistance to diverse BLS strains in the heirloom cultivar Carolina Gold Select	[93]
***Bs2***	Pepper	*X. euvesicatoria* (bacterial spot xanthomonads)	NLR-mediated recognition of *AvrBs2* (classic gene-for-gene ETI)	Strong race-specific resistance when *AvrBs2* present	[20]
***Bs3***	Pepper	*X. euvesicatoria*	Executor gene triggering HR upon AvrBs3 binding	Race-specific resistance	[125,126]
***Rx3***	Tomato	*X. euvesicatoria*	ETI-associated resistance locus (tomato bacterial spot resistance)	Resistance to specific race(s); used in breeding	[122]
***Rx4***	Tomato	*X. euvesicatoria* pv. *perforans*	ETI-associated hypersensitive response to race T3	Strong race-specific resistance	[123]
***Roq1* (transferable *NLR*)**	*Nicotiana* (and engineered solanaceous crops)	*Xanthomonas* (XopQ)	NLR recognition of conserved effector XopQ (broad ETI; transgene utility)	Broad resistance to multiple strains carrying XopQ	[186,202]
**Quantitative/QTL-based resistance (*Brassica*)**	*B. napus*/*B. oleracea*	*X. campestris* pv. *campestris*	Polygenic resistance with defense/metabolic reprogramming (quantitative resistance)	Partial but potentially durable reduction in black rot	[115,117,118]
***CsLOB1*** **promoter edits**	Citrus	*X. citri* subsp. *citri*	CRISPR editing disrupts TALE (PthA4) binding sites; blocks canker development	Broad-spectrum resistance in transgenic lines	[101,102]
***SWEET* EBE promoter editing**	Rice	*X. oryzae* pv. *oryzae*	Genome editing removes multiple TALE EBEs in SWEET promoters (engineered S-gene resistance)	Broad-spectrum resistance without major yield penalty	[86,87,88,209,210]

**Table 5:** Sustainable Management Strategies Against *Xanthomonas* spp.

Strategy	Mechanism	Examples	Efficacy	References
**Biocontrol agents**	Antagonize pathogens via antimicrobial metabolites	*Bacillus amyloliquefaciens*, *Pseudomonas* spp.	50–80% reduction in disease severity	[229,230]
**CRISPR-based resistance**	Edit host susceptibility genes (e.g., *SWEET14*, *CsLOB1*)	Rice *SWEET14* edits	Broad-spectrum resistance without yield loss	[87,102]
**Nanoparticle antimicrobials**	Disrupt bacterial membranes or deliver targeted copper/zinc ions	Cu-Zn nanoparticles, chitosan formulations	Overcomes copper resistance; eco-friendly	[239,241]
**ISR**	Prime plant immunity via SA/JA signaling pathways	*Streptomyces* spp., chumacin QS inhibitors	Up to 70% symptom reduction	[235,236]

## Data Availability

Not applicable.
